# New Pythagorean Entropy Measure with Application in Multi-Criteria Decision Analysis

**DOI:** 10.3390/e23121600

**Published:** 2021-11-29

**Authors:** Neeraj Gandotra, Bartłomiej Kizielewicz, Abhimanyu Anand, Aleksandra Bączkiewicz, Andrii Shekhovtsov, Jarosław Wątróbski, Akbar Rezaei, Wojciech Sałabun

**Affiliations:** 1 Yogananda School of AI, Computers and Data Science, Shoolini University, Solan 173229, Himachal Pradesh, India; neerajgandotra@shooliniuniversity.com (N.G.); anand45510@gmail.com (A.A.); 2 Research Team on Intelligent Decision Support Systems, Department of Artificial Intelligence and Applied Mathematics, Faculty of Computer Science and Information Technology, West Pomeranian University of Technology in Szczecin, ul. Żołnierska 49, 71-210 Szczecin, Poland; bartlomiej-kizielewicz@zut.edu.pl (B.K.); andrii-shekhovtsov@zut.edu.pl (A.S.); 3 Institute of Management, University of Szczecin, ul. Cukrowa 8, 71-004 Szczecin, Poland; aleksandra.baczkiewicz@phd.usz.edu.pl (A.B.); jaroslaw.watrobski@usz.edu.pl (J.W.); 4 Doctoral School of University of Szczecin, ul. Mickiewicza 16, 70-383 Szczecin, Poland; 5 Department of Mathematics, Payame Noor University, Tehran P. O. Box 19395-3697, Iran; rezaei@pnu.ac.ir

**Keywords:** Pythagorean fuzzy sets, entropy measures, Pythagorean fuzzy entropy, multi-criteria decision analysis, MCDA, COPRAS method

## Abstract

The purpose of this paper is to propose a new Pythagorean fuzzy entropy for Pythagorean fuzzy sets, which is a continuation of the Pythagorean fuzzy entropy of intuitionistic sets. The Pythagorean fuzzy set continues the intuitionistic fuzzy set with the additional advantage that it is well equipped to overcome its imperfections. Its entropy determines the quantity of information in the Pythagorean fuzzy set. Thus, the proposed entropy provides a new flexible tool that is particularly useful in complex multi-criteria problems where uncertain data and inaccurate information are considered. The performance of the introduced method is illustrated in a real-life case study, including a multi-criteria company selection problem. In this example, we provide a numerical illustration to distinguish the entropy measure proposed from some existing entropies used for Pythagorean fuzzy sets and intuitionistic fuzzy sets. Statistical illustrations show that the proposed entropy measures are reliable for demonstrating the degree of fuzziness of both Pythagorean fuzzy set (PFS) and intuitionistic fuzzy sets (IFS). In addition, a multi-criteria decision-making method complex proportional assessment (COPRAS) was also proposed with weights calculated based on the proposed new entropy measure. Finally, to validate the reliability of the results obtained using the proposed entropy, a comparative analysis was performed with a set of carefully selected reference methods containing other generally used entropy measurement methods. The illustrated numerical example proves that the calculation results of the proposed new method are similar to those of several other up-to-date methods.

## 1. Introduction

Entropy is a measure of uncertainty occurring in information in which a higher value implies more information in the process being analyzed. In multi-criteria decision analysis (MCDA) methods, entropy is used to determine the objective weights of the criteria based on the data in the decision matrix. The entropy of a Pythagorean fuzzy set is a novel approach that measures the uncertainty associated with a PFS that represents the properties of fuzzy sets well. The entropy designed for fuzzy sets measures fuzziness appearing among fuzzy sets [1]. Multi-criteria decision-making processes are often fraught with uncertainty due to constraints of knowledge regarding the choice of criteria for the decision model and its structure, inaccuracies present in the input data, and limited stakeholders’ competence [2]. Pythagorean fuzzy entropy is a novel, flexible tool for handling inaccurate information in multi-criteria decision problems incorporating uncertainty [3]. Several extensions of fuzzy set theory are present in the literature and are utilized in the domain of decision making. A Pythagorean fuzzy set is a generalization of an intuitionistic fuzzy set. Atanassov proposed intuitionistic fuzzy sets in 1983. An intuitionistic fuzzy set must hold for 0≤μ+ϑ≤1 where $\mu \to \left[0,1\right]$ and $\vartheta \to \left[0,1\right]$. For example, for the values μ=0.8,ϑ=0.3 the intuitionistic fuzzy set condition fails, so for such scenario, Pythagorean fuzzy sets are used; they were proposed by Yager in 2013 [4] and have membership and non-membership grade such that it should satisfy the condition $0\leq \mu^{2}+\vartheta^{2}\leq 1$, where values lie in the interval [0,1]. Pythagorean fuzzy sets are an extension of intuitionistic fuzzy sets introduced previously by Atanassov [5,6]. Pythagorean fuzzy sets and intuitionistic fuzzy sets are closely related. With their application, experts have more flexibility to make judgments considering uncertainties and ambiguities in the risk assessment problem [4]. The correlation coefficient plays a significant role in decision making, analyzing data, etc. [7]. Furthermore, Tadeusz Gerstenkorn and Jacek Manko coined a correlation coefficient in intuitionistic fuzzy sets [8]. First, in 2016, Garg gave the concept of correlation coefficient in Pythagorean fuzzy sets. From a statistical point of view, the correlation coefficient is defined as the power of the linear correlation between two random variables [8]. Again, from a statistical point of view, the domain of the correlation coefficient is [−1,1]. However, the research that Xuan Thao and other studies put forward concluded that a more appropriate domain value for the correlation coefficient is [0,1]. Zhang and Xu introduced the concept of a Pythagorean fuzzy number [9]. In decision making, the Pythagorean fuzzy model has more application range than the intuitionistic fuzzy model. In various real-life aspects, Pythagorean fuzzy sets are used. For example, Pythagorean fuzzy entropy is used in mine factories for the assessment of life safety risk [10]. Fuzzy and probability theories are related, but the critical difference between probability and fuzzy theories is that we deal with random events that may occur or not in probability theory. However, fuzzy logic deals with truth values only. Various researchers have put forward different multi-criteria decision making (MCDM). For example, a student may need to consider various university parameters before taking admission. Companies or individuals do not depend on one criterion for their decision. They choose multiple criteria for their decision. There exist various decision problems such as sorting problems or ranking problems. Sometimes, decision making becomes very complex. It is applied in many fields such as the financial sector, education sector, etc. The COPRAS method was introduced by Zavadskas in 1994 [11]. In the COPRAS method, there exist *m* alternatives and *n* criteria; first we find entropies equal to the criteria, then we calculate the weight criteria. In step 3, we create a decision matrix and all the steps are explained in detail in this paper in the example section. There are various other multi-criteria decision-making methods. In early 2010, Balezentis proposed one in [12]. The focus of this article was on sustainable development in the European Union. In this research paper, we will work on the complex proportional assessment (COPRAS) MCDM method. Different researchers have put forward different entropies in different fields. In this paper, we put forward an entropy with the complex proportional assessment (COPRAS) method.

The rest of the paper is organized as follows. Section 2 provides a review of the literature focused on Pythagorean fuzzy sets, Pythagorean fuzzy entropy and the COPRAS method. In Section 3, basic concepts and assumptions of intuitionistic fuzzy sets and Pythagorean fuzzy sets are included. Section 4 gives the fundamentals of the novel Pythagorean fuzzy entropy measure. In Section 5, Pythagorean fuzzy multi-criteria decision making based on COPRAS is explained in subsequent stages. Section 6 provides a comparison of entropies proposed in this paper. In Section 7, the case study is introduced, and results are presented and discussed. Finally, in Section 8 conclusions are drawn.

## 2. Literature Review

Multi-criteria decision analysis (MCDA) methods have been developed to support decision making that requires consideration of multiple, often conflicting, criteria. Unfortunately, humans have limited capabilities to process information containing large amounts of data. Therefore, decision making based on intuition with a selective consideration of criteria is usually insufficient. MCDA algorithms allow structuring the decision problem by handling all data and criteria and each criterion’s importance. Many MCDA methods based on different algorithms are available, each of which considers and structures the data differently [13].

MCDA methods are developed within two main streams, the American school and the European school. The methods of the American school are based on the functional strategy, represented by the utility function. These methods take into account indifference and preference between the evaluated alternatives and do not consider their incomparability [14]. This group of methods includes analytic hierarchy process (AHP), technique for order of preference by similarity to ideal solution (TOPSIS), Vise Kriterijumska Optimizacija I Kompromisno Resenje (VIKOR), multi-attribute utility theory (MAUT), simple multi-attribute rating technique (SMART), measuring attractiveness by a categorical based evaluation technique (MACBETH), and complex proportional assessment (COPRAS). Furthermore, methods coming from the American school usually do not consider uncertainties and inaccuracies that occur in the data under evaluation several times. Instead, this group of methods strongly incorporates a strategy using a single synthesized criterion [15].

Methods from the European school use a relational model that considers indifference, weak or strong preference, and incomparability. These methods aggregate preferences using a ranking relation that is not transitive between pairs of alternatives. The most popular methods from the European school are the elimination and choice expressing the reality (ELECTRE) family, preference ranking organization method for enrichment evaluations (PROMETHEE), novel approach to imprecise assessment and decision environments (NAIADE), and treatment of the alternatives according to the importance of criteria (TACTIC). Besides the two main streams described above, there is a group of mixed and rule-based methods. Mixed methods integrate the approaches of the American and European schools, such as the EVAMIX method, which makes it possible to take into account the quantitative and qualitative types of the criteria considered, using two different measures of dominance [16]. The rule-based method that is the characteristic objects method (COMET) works on the triangular fuzzy numbers provided for each criterion, which determine the degree to which the alternatives belong to the given linguistic values defining the criteria under consideration. The generated characteristic objects are then compared pairwise [17]. Together with the aggregated ranking values, the alternatives create a fuzzy rule base that is activated in evaluating the alternatives [18].

The lack of consideration of volatility, inaccuracy, and uncertainty in expert assessments is the main reason for criticism of methods from the American school by researchers representing the European school of MCDA [14]. In an effort to eliminate the limitations mentioned above, the methods from the American school are being developed. It started from the resignation of the simple aggregation, through their developments, including fuzzy versions and fuzzy sets. As a result, these methods give more reliable results in solving problems where inaccuracies and uncertainties are present. In this work, the authors propose an extension of the COPRAS method derived from the American school with Pythagorean entropy used to determine the significance of the criteria considered in evaluating alternatives expressed in terms of weights. This approach makes it possible to adequately account for the uncertainties and imprecisions present in most decision problems and thus obtain more robust final results.

### 2.1. The COPRAS Method

The complex proportional assessment (COPRAS) method was developed by Zavadskas and Kaklauskas in 1994 [19]. This method belongs to the American MCDA school, similar to TOPSIS, VIKOR, and AHP. The significance of the alternatives investigated is proportionally dependent on the weights of the criteria that define them [15]. The COPRAS algorithm uses a staged ranking and evaluation of alternatives concerning the importance and grade of the utility expressed in the criteria weights [20]. This method is used to evaluate the advantage of one alternative over another and to benchmark alternatives. Data normalization techniques are used in this methodology, which can produce very different results [21]. The COPRAS algorithm is helpful for both maximizing and minimizing criteria, provided that at least two different criteria are considered [22]. The concept of the COPRAS algorithm is similar to that of the simple additive weighing (SAW) method for maximizing criteria. However, for minimizing criteria, COPRAS uses its proprietary transformation of the method [23]. A particular advantage of COPRAS is the accuracy of the provided rankings of alternatives due to the separate evaluation of profit and cost criteria [24]. Thus, the final COPRAS score is derived from the positive ideal solution and the negative ideal solution, and the best alternative should have the highest value for the positive ideal solution and the lowest value for the negative ideal solution [25,26].

Recent literature review shows that the COPRAS method is readily and widely used to solve multi-criteria decision problems. For example, COPRAS was used to assess the safety of regions with COVID-19 [22], for evaluation of energy sectors sustainability [11], for supplier selection in the oil and gas industry with uncertainty, utility functions, and criteria representing performance [27]. In another study, COPRAS was combined with AHP and applied to the construction industry to assess the performance of the variant design of a timber–concrete composite (TCC) floor system [28]. The AHP method was used in that paper to determine the significance of the criteria. In turn, COPRAS was combined with the criteria importance through inter-criteria correlation (CRITIC) weighting technique in [29] dedicated to assessing the performance of companies in aspects of intelligent and sustainable industrial technologies. The same combination was applied to the electric motorcycle evaluation in [30]. Fuzzy COPRAS was used for multi-criteria selection of optimum material to improve braking system in the automotive industry, to ensure maximum safety and failure-free performance [31]. The authors of [32] presented the application of the COPRAS method in combination with the objective entropy technique to determine criteria weights in the problem of selecting conveying equipment in the industry sector. The references presented above prove the usefulness of the COPRAS method in multi-criteria problems in various domains, including those in which uncertainty occurs and in engineering problems in which the appropriate decision has an impact on safety and a lack of emergency performance. The incorporated COPRAS approach based on Pythagorean fuzzy sets visibly demonstrates the ability to determine the optimal solution and provides more accurate information than other approaches [3].

### 2.2. Intuitionistic Fuzzy Sets

The attempt to adapt and develop methods for multi-criteria decision making in a fuzzy environment became the motivation for Atanassov to introduce intuitionistic fuzzy sets (IFSs) [33], which represent fuzzy properties of objects in an easy-to-understand way. IFSs represent an extension of fuzzy sets (FSs) [34]. An IFS satisfies the requirement that the sum of the degrees of membership and non-membership is equal to 1 [35]. IFSs allow for easy modeling of both symmetrical and asymmetrical linguistic values and more accurate expression of information [36]. IFSs find wide application in real-world risk evaluation procedures with ambiguity and uncertainty. IFSs can be combined with MCDA methods such as AHP, COMET and potentially all pairwise rankings of all possible alternatives (PAPRIKA) method [36,37]. However, the ability to model uncertainty and imprecision provided by IFS theory is constrained. Therefore, Pythagorean fuzzy sets can catch and model more uncertain information for complex problems occurring in the real world [6].

### 2.3. Pythagorean Fuzzy Sets

The idea of Pythagorean fuzzy sets demonstrates a superiority over well-established fuzzy sets like intuitionistic and interval-valued ones when problems with uncertainty and ambiguity need to be solved [6]. Pythagorean fuzzy sets (PFS) are beneficial in compound, real-world situations because they can model more uncertain and subjective information not handled by intuitionistic fuzzy sets. Due to their valuable properties, PFS is often used in conjunction with MCDA methods to solve decision problems with uncertainty. In addition, computer systems based on fuzzy sets are often used in several domains, such as risk assessment, to cope with accurate danger estimation. For example, the authors of [4] used the VIKOR method extended by Pythagorean fuzzy sets to define hazards and risks in constructing a natural gas pipeline. The authors of [6] presented an application of Pythagorean fuzzy information in combination with VIKOR as well. In another work, the PF and PROMETHEE II based approach was used in the construction industry to solve the problem of selecting bridge construction methods under complex uncertainty [38]. TOPSIS has been combined with PFS in green supplier selection for green supply chain management in sustainable development in [39]. With PFS’s thoughtful consideration of uncertainty, it is possible to accurately frame this problem as a complex process that includes installation, supply, procurement, deliveries, production, and waste management.

PFS with Pythagorean membership degrees fulfills the requirement that the square sum of the membership degree and the non-membership degree is equal to or less than 1 [40]. Furthermore, the approach uses uncertain information expressed by Pythagorean fuzzy values and creates the terms such as generalized distance measure and distance indices. Pythagorean fuzziness theory contributed to the growing popularity of PFSs after the introduction of the mathematical representation of PFS by Zhang and Xu in 2014 [9].

### 2.4. Pythagorean Fuzzy Entropy

In MCDA problems, weights are essential since their values assigned to the criteria determine the final result of the evaluation of alternatives. However, the relevance of the criteria is not known for every problem. Such a situation occurs when the decision-maker does not sufficiently know the problem being solved to prioritize the criteria. Therefore, it is necessary to use objective weighting methods based on mathematical algorithms and formulas and determine weights based on the information contained in the input data. For entirely unknown information about the weights in the MCDA problem, entropy is recommended [41].

Entropy is a measure of uncertain or fuzzy information. Pythagorean fuzzy entropy (PFE) is used to express fuzziness and uncertainty occurring in PFS. The PFE includes the part represented by the similarity between the PFS and its supplement and from the hesitancy part [1,10]. Higher entropy indicates more information in the process. Decision making incorporating a measure of entropy is beneficial because a high entropy value represents low uncertainty [42]. Fuzzy entropy was primarily introduced by Zadeh [43]. The axiomatic construction of fuzzy set entropy was introduced by De Luca and Termini regarding Shannon’s probability entropy [44]. Hung and Yang extended this idea to the idea of fuzzy set entropy in IFSs [45]. The development of IFS was a motivation for further research in the entropy domain. In 1996, Burille and Bustince proposed an axiomatically defined entropy for IFS [46]. Szmidt and Kacprzyk introduced entropy for intuitionistic fuzzy sets in 2001 using a geometric interpretation of IFS, specifically the ratio of the distances among them [47]. In 2016, Zhu and Li presented new axioms for the entropy measure in IFSs [48]. It can be observed that entropy for intuitionistic fuzzy sets and Pythagorean fuzzy sets finds application in the practical solution of problems affected by uncertainty from various domains. For example, a new entropy measure using Pythagorean fuzzy sets integrated with the COPRAS method was applied to solve the problem of component supplier selection for fuel cell combined with hydrogen technology [3]. On the other hand, the Pythagorean fuzzy analytical hierarchical process (PFAHP) method, along with information entropy, has been applied to evaluate the significance of the weights of the criteria defining security in software defined network (SDN), in the SDN architecture risk assessment problem [49].

However, this concept has limitations when applied to Pythagorean fuzzy sets (PFSs), because in several instances, they do not satisfy the required, acceptable performance. Therefore, for PFSs, Pythagorean fuzzy entropy is appropriate, which extends the axioms in Hung and Yang’s idea of fuzzy entropy [50]. This measure is simple, close to statistical relevance, and adequately represents fuzzy attributes [51].

Pythagorean fuzzy set theory reflects the fuzziness of the objective world more effectively than IFS theory. Thus, many researchers are focused on PFE investigations. In 2018, Yang and Hussain introduced four measures of Pythagorean Entropy founded on probability, distance, Pythagorean index, and a minimum-maximum technique [1]. Xue et al. proposed a new entropy measure for PFSs based on similarity and hesitancy [10]. Wan et al. introduced an entropy measure for PFSs founded on the axiomatic definition of Szmidt and Kacprzyk [52]. Peng et al. [53] proposed 12 entropies for PFSs, relying on the axiomatic definition of Burille and Bustince.

## 3. Fundamental Concepts

### 3.1. Intuitionistic Fuzzy Set

**Definition** **1** ([54])**.** *An intuitionistic fuzzy set ξ in a finite universe* Φ *is an object that the following notation can characterize:*

(1) $$\xi =\left\{\langle y,\mu_{\xi }\left(y\right),\nu_{\xi }\left(y\right)|y\in \Phi \right\}$$

*where the functions $\mu_{\xi }\left(y\right)\in \Phi \to \left[0,1\right]$ and $\nu_{\xi }\left(y\right)\in \Phi \to \left[0,1\right]$ express the membership degree and non-membership degree of y to ξ in *Φ*, respectively. Given any element y in *Φ*, the following is accomplished:*

(2) $$0\leq \mu_{\xi }\left(y\right)+\nu_{\xi }\left(y\right)\leq 1$$

*The function $\lambda_{\xi }\left(y\right)$, denominated as the hesitation degree, which expresses insufficient information concerning whether y belongs to ξ in:*

(3) $$\lambda_{\xi }\left(y\right)=1-\mu_{\xi }\left(y\right)-v_{\xi }\left(y\right)$$

### 3.2. Pythagorean Fuzzy Set

**Definition** **2** ([4])**.** *A Pythagorean fuzzy set ξ in a finite universe* Φ *is an object that the following notation can characterize:*

(4) $$\xi =\left\{\langle y,\mu_{\xi }\left(y\right),\nu_{\xi }\left(y\right)|y\in \Phi \right\}$$

*where the functions $\mu_{\xi }\left(y\right)\in \Phi \to \left[0,1\right]$ and $\nu_{\xi }\left(y\right)\in \Phi \to \left[0,1\right]$ express the membership degree and non-membership degree of y to ξ in *Φ*, respectively. Given any element y in *Φ*, the following is accomplished:*

(5) $$0\leq \mu_{\xi }^{2}\left(y\right)+\nu_{\xi }^{2}\left(y\right)\leq 1$$

*The function $\lambda_{\xi }\left(y\right)$, denominated as the hesitation degree, which expresses insufficient information concerning whether y belongs to ξ in, is expressed as follows:*

(6) $$\lambda_{\xi }\left(y\right)={\left[1-{\left(\mu_{\xi }\left(y\right)\right)}^{2}-{\left(v_{\xi }\left(y\right)\right)}^{2}\right]}^{\frac{1}{2}}$$

*For computational suitability Zhang and Xu [9] defined $\left(\mu_{\xi }\left(y\right),\nu_{\xi }\left(y\right)\right)$ as a Pythagorean fuzzy number and denoted it by $\xi =(\mu_{\xi },\nu_{\xi })$.*

The basic operations of Pythagorean fuzzy numbers are defined by Zhang and Xu [9] as follows:

**Definition** **3** ([9])**.** *Let $\xi =(\mu_{\xi }\left(y\right),\nu_{\xi }\left(y\right))$ and $\psi =(\mu_{\psi }\left(y\right),\nu_{\psi }\left(y\right))$ be two Pythagorean fuzzy numbers and λ>0, then their basic operations are as follows:*

- *$\xi \subset \psi \;\;if\;\;\forall y\in \Phi ,\mu_{\xi }\left(y\right)\leq \mu_{\psi }\left(y\right)\;\;and\;\;\nu_{\xi }\left(y\right)\geq \nu_{\psi }\left(y\right)$;*
- $\xi^{c}=\bar{\xi }=\left\{\langle y,v_{\xi }\left(y\right),\mu_{\xi }\left(y\right)\rangle |y\in \Phi \right\}$
- *ξ=ψifξ⊂ψ and ψ⊂ξ*
- *$\xi \cap \psi =\left\{\langle y,min\left(\mu_{\xi }\left(y\right),\mu_{\psi }\left(y\right)\right),max\left(\nu_{\xi }\left(y\right),\nu_{\psi }\left(y\right)\right)\rangle |y\in \Phi \right\}$;*
- $\xi \cup \psi =\left\{\langle y,max\left(\mu_{\xi }\left(y\right),\mu_{\psi }\left(y\right)\right),min\left(\nu_{\xi }\left(y\right),\nu_{\psi }\left(y\right)\right)\rangle |y\in \Phi \right\};$
- $\xi ⊕\psi =\left\{\langle y,\sqrt{\mu_{\xi }^{2}\left(y\right)+\mu_{\psi }^{2}\left(y\right)-\mu_{\xi }^{2}\left(y\right)\mu_{\psi }^{2}\left(y\right)},\nu_{\xi }\left(y\right)\nu_{\psi }\left(y\right)\rangle |y\in \Phi \right\};$
- $\xi ⊗\psi =\left\{\langle y,\mu_{\xi }\left(y\right)\nu_{\psi }\left(y\right),\sqrt{\nu_{\xi }^{2}\left(y\right)+\nu_{\psi }^{2}\left(y\right)-\nu_{\xi }^{2}\left(y\right)\nu_{\psi }^{2}\left(y\right)},\rangle |y\in \Phi \right\};$
- $\lambda \xi =\left\{y,\left(\sqrt{1-{\left(1-\mu_{\xi }^{2}\left(y\right)\right)}^{\lambda }},\nu_{\xi }{\left(y\right))}^{\lambda }\right)|y\in \Phi \right\};$
- $\xi^{\lambda }=\left\{y,\left({\left(\mu_{\xi }\left(y\right)\right)}^{\lambda },\sqrt{1-{\left(1-\nu_{\xi }^{2}\left(y\right)\right)}^{\lambda }}\right)|y\in \Phi \right\}.$

## 4. Novel Pythagorean Fuzzy Entropy Measure

The proposed entropy, as opposed to the method proposed for Pythagorean fuzzy sets by Xue, Xu, Zhang, and Tian [10], gives different results, which are presented in Section 6. These results contribute to different weight values in multi-criteria decision making with uncertain data. An aspect of this would need to be considered more extensively in future research for more complex problems. Our innovation is also applying a flexibility secant function to the entropy, adapted to give more nonlinear responses. The use of such solutions is favorable to the present trends related to the development of tools that are adapted to handle nonlinearity, e.g., in multi-criteria decision analysis [55,56]. An innovative entropy measure for a PFS is determined by the notion of Wang and Wang [57] in the context of intuitionistic fuzzy sets as follows:

**Definition** **4.** *For the Pythagorean fuzzy set $\xi =\left\{\langle y,\mu_{\xi }\left(y\right),\nu_{\xi }\left(y\right)|y\in \Phi \right\}$, the Pythagorean fuzzy entropy of ξ is defined as follows:*

(7) $$E\left(\xi \right)=\frac{1}{n}\sum_{i=1}^{n}\left[sec\left(\frac{\pi }{3}-\frac{\left|\mu_{\xi }^{2}\left(y\right)-\nu_{\xi }^{2}\left(y\right)\right|}{3}\pi \right)-1\right]$$

### Properties for Pythagorean Fuzzy Entropy Measure

**Definition** **5.** *The mapping E:PFS(Φ)→[0,1] is stated as a Pythagorean fuzzy entropy if it satisfies:*

*1.*  *$E\left(\xi \right)=0$ if and only if ξ is a crisp set;*
*2.*  *$E\left(\xi \right)=1$ if and only if $\mu_{\xi }\left(y\right)=\nu_{\xi }\left(y\right)=\frac{3\sqrt{3}}{9},\forall y\in \Phi$;*
*3.* 
$E\left(\psi \right)\leq E\left(\xi \right)\;if\;\psi \;is\;\;less\;fuzzy\;than\;\xi ,\;i.e.,\;\mu_{\psi }\left(y\right)\leq \mu_{\xi }\left(y\right)\;and\;\nu_{\xi }\left(y\right)\leq \nu_{\psi }\left(y\right)\;for\;\mu_{\xi }\left(y\right)\leq \nu_{\xi }\left(y\right)\;or\;\mu_{\psi }\left(y\right)\geq \mu_{\xi }\left(y\right)\;and\;\nu_{\xi }\left(y\right)\geq \nu_{\psi }\left(y\right)\;for\;\mu_{\xi }\left(y\right)\geq \nu_{\xi }\left(y\right),\forall y\in \Phi ;$

*4.* 
$E\left(\xi \right)=E\left(\xi^{c}\right),\forall \xi \in PFS\left(\Phi \right).$

**Theorem** **1.** *$E\left(\xi \right)$ is a Pythagorean fuzzy entropy.*

*Proof: First, we should prove $0\leq E\left(\xi \right)\leq 1.$ Since $0\leq \mu_{\xi }^{2}\left(y\right),\nu_{\xi }^{2}\left(y\right)\leq 1$ and $0\leq \mu_{\xi }^{2}\left(y\right)+\nu_{\xi }^{2}\left(y\right)\leq 1,\left|\mu_{\xi }^{2}\left(y\right)-\nu_{\xi }^{2}\left(y\right)\right|=\left|\left(\mu_{\xi }\left(y\right)+\nu_{\xi }\left(y\right)\right)\times \left(\mu_{\xi }\left(y\right)-\nu_{\xi }\left(y\right)\right)\right|$.*

*Then,$\frac{\pi }{3}\left|\mu_{\xi }^{2}\left(y\right)-\nu_{\xi }^{2}\left(y\right)\right|\leq \frac{\pi }{3}$. Thus, $0\leq E\left(\xi \right)\leq 1$.*

*Next, $E\left(\xi \right)$ meets the four properties of Pythagorean fuzzy entropy, which are proved as follows:*

*1.*  *If $E\left(\xi \right)=0$, then $\frac{\pi }{3}\left|\mu_{\xi }^{2}\left(y\right)-\nu_{\xi }^{2}\left(y\right)\right|=\frac{\pi }{3}$. We have $\mu_{\xi }\left(y\right)=0,\nu_{\xi }\left(y\right)=1,\pi_{\xi }\left(y\right)=0$ or $\mu_{\xi }\left(y\right)=1,\nu_{\xi }\left(y\right)=0,\pi_{\xi }\left(y\right)=0$. Hence ξ is a crisp set. If ξ is a crisp set, then $E\left(\xi \right)=0$.*
*2.*  *If $E\left(\xi \right)=1$, then $\frac{\pi }{3}\left|\mu_{\xi }^{2}\left(y\right)-\nu_{\xi }^{2}\left(y\right)\right|=0$. We have $\mu_{\xi }\left(y\right)-\nu_{\xi }\left(y\right)=0$, namely, $\mu_{\xi }\left(y\right)=\nu_{\xi }\left(y\right)$.*
*If $\mu_{\xi }\left(y\right)=\nu_{\xi }\left(y\right)$, then $E\left(\xi \right)=1$.*
*3.*  *Construct a function, $f\left(\alpha ,\beta \right)=sec\left(\frac{\pi }{3}-\frac{\left|\alpha^{2}-\beta^{2}\right|}{3}\pi \right)-1$, where α,β∈[0,1].*
*Case 1: when α≤β, then $f_{1}\left(\alpha ,\beta \right)=sec\left(\frac{\pi }{3}-\frac{\left|\beta^{2}-\alpha^{2}\right|}{3}\pi \right)-1$.*
*We need to prove that $f_{1}\left(\alpha ,\beta \right)$ increases with α and decreases with β*
*Now, $\frac{\partial f_{1}\left(\alpha ,\beta \right)}{\partial \alpha }=\frac{2\alpha \pi }{3}\left[sec\left(\frac{\pi }{3}-\frac{\left|\beta^{2}-\alpha^{2}\right|}{3}\pi \right)tan\left(\frac{\pi }{3}-\frac{\left|\beta^{2}-\alpha^{2}\right|}{3}\pi \right)\right]$ and*
*$\frac{\partial f_{1}\left(\alpha ,\beta \right)}{\partial \beta }=-\frac{2\beta \pi }{3}\left[sec\left(\frac{\pi }{3}-\frac{\left|\beta^{2}-\alpha^{2}\right|}{3}\pi \right)tan\left(\frac{\pi }{3}-\frac{\left|\beta^{2}-\alpha^{2}\right|}{3}\pi \right)\right]$.*
*When $\alpha \leq \beta ,\frac{\partial f_{1}\left(\alpha ,\beta \right)}{\partial \alpha }\geq 0$ and $\frac{\partial f_{1}\left(\alpha ,\beta \right)}{\partial \beta }\leq 0$, then we can say that $f_{1}\left(\alpha ,\beta \right)$ increases with α and decreases with β, then $\mu_{\psi }\left(y\right)\leq \nu_{\psi }\left(y\right)$ and $\mu_{\xi }\left(y\right)\leq \mu_{\psi }\left(y\right),\nu_{\xi }\left(y\right)\geq \nu_{\psi }\left(y\right)$ are satisfied.*
*Thus, we have $f\left(\mu_{\xi }\left(y\right),\nu_{\xi }\left(y\right)\right)\leq f\left(\mu_{\psi }\left(y\right),\nu_{\psi }\left(y\right)\right)$.*
*Case 2: When α≥β, then $f_{2}\left(\alpha ,\beta \right)=sec\left(\frac{\pi }{3}-\frac{\left|\alpha^{2}-\beta^{2}\right|}{3}\pi \right)-1$.*
*We need to prove that $f_{2}\left(\alpha ,\beta \right)$ decreases with α and increases with β.*
*Now, $\frac{\partial f_{2}\left(\alpha ,\beta \right)}{\partial \alpha }=-\frac{2\alpha \pi }{3}\left[sec\left(\frac{\pi }{3}-\frac{\left|\alpha^{2}-\beta^{2}\right|}{3}\pi \right)tan\left(\frac{\pi }{3}-\frac{\left|\alpha^{2}-\beta^{2}\right|}{3}\pi \right)\right]$ and*

$\frac{\partial f_{2}\left(\alpha ,\beta \right)}{\partial \beta }=\frac{2\beta \pi }{3}\left[sec\left(\frac{\pi }{3}-\frac{\left|\alpha^{2}-\beta^{2}\right|}{3}\pi \right)tan\left(\frac{\pi }{3}-\frac{\left|\alpha^{2}-\beta^{2}\right|}{3}\pi \right)\right].$

*When $\alpha \geq \beta ,\frac{\partial f_{2}\left(\alpha ,\beta \right)}{\partial \alpha }\leq 0$ and $\frac{\partial f_{2}\left(\alpha ,\beta \right)}{\partial \beta }\geq 0$, then we can say that $f_{2}\left(\alpha ,\beta \right)$ decreases with α and increases with β, then $\mu_{\psi }\left(y\right)\geq \nu_{\psi }\left(y\right)$ and $\mu_{\xi }\left(y\right)\geq \mu_{\psi }\left(y\right),\nu_{\xi }\left(y\right)\leq \nu_{\psi }\left(y\right)$ are satisfied.*
*Thus, we have $f\left(\mu_{\xi }\left(y\right),\nu_{\xi }\left(y\right)\right)\leq f\left(\mu_{\psi }\left(y\right),\nu_{\psi }\left(y\right)\right)$.*
*Hence ξ≤ψ, then we can say that $E\left(\xi \right)\leq E\left(\psi \right)$.*
*4.*  *$E\left(\xi \right)=\frac{1}{n}\sum_{i=1}^{n}\left[sec\left(\frac{\pi }{3}-\frac{\left|\mu_{\xi }^{2}\left(y\right)-\nu_{\xi }^{2}\left(y\right)\right|}{3}\pi \right)-1\right]$;*
*5.*  *$E\left(\xi^{c}\right)=\frac{1}{n}\sum_{i=1}^{n}\left[sec\left(\frac{\pi }{3}-\frac{\left|\nu_{\xi }^{2}\left(y\right)-\mu_{\xi }^{2}\left(y\right)\right|}{3}\pi \right)-1\right]$.*

## 5. Pythagorean Fuzzy Multi-Criteria Decision Making Based on COPRAS

**Figure 1:**
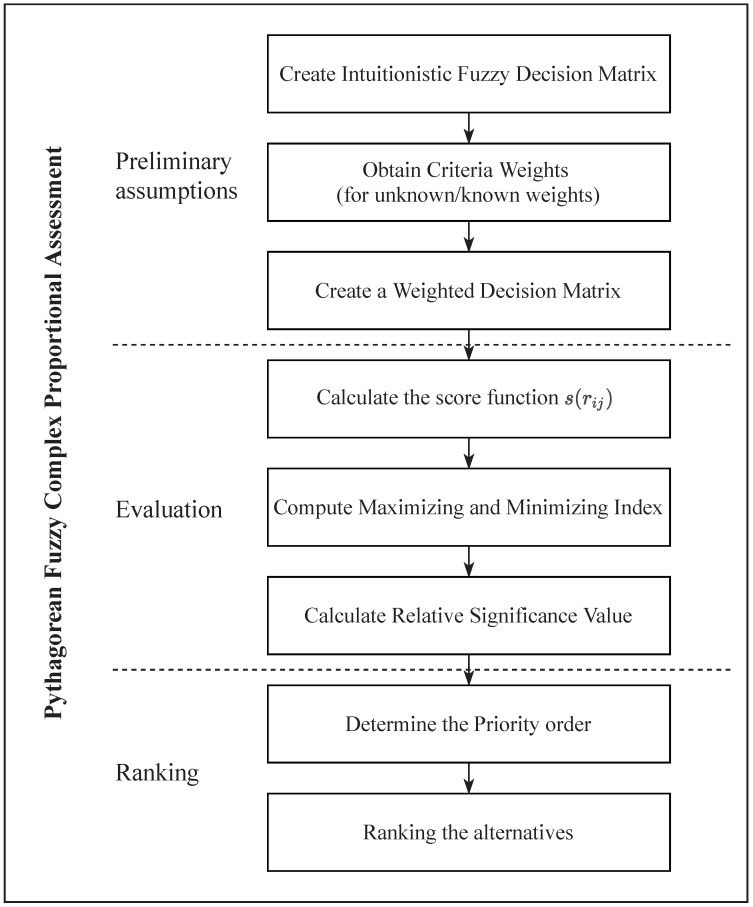
Subsequent stages of COPRAS method.

The COPRAS technique, initiated by Zavadskas and Kaklauskas in 1994 [19], is a fast developed method to deal with real problems and multi-criteria decision making. We suggest the complex proportional assessment (COPRAS) method for multiple criteria decision-making problems in this section. It is based on the proposed innovative entropy measure. Let $A=\left\{A_{1},A_{2},\ldots ,A_{m}\right\}$ be a set of alternatives. We must aim at the best selection based on the set of criteria $C=\left\{C_{1},C_{2},\ldots ,C_{n}\right\}$. Given the Pythagorean fuzzy decision matrix $D={\left[d_{ij}\right]}_{m\times n}$, where $d_{ij}=\left(\mu_{ij},\nu_{ij}\right)$ is a Pythagorean number displaying the performance of value of alternative $A_{i}$ on the criterion $C_{j}$. The algorithm of the COPRAS method is represented in Figure 1. On the other hand, the structure of the whole study can be shown in the following steps [51]:

**Step** **1.** Compute the entropy on Pythagorean fuzzy sets $e_{j}$ of the criterion $C_{j\;}\left(j=1,2,\ldots ,n\right)$.
**Step** **2.** Calculate the weight $w_{j}$ of the criterion $C_{j\;}\left(j=1,2,\ldots ,n\right)$ by using the expression:
  (8) $$w_{j}=\frac{1-e_{j}}{\sum_{j=1}^{n}\left(1-e_{j}\right)},\;\left(j=1,2,\ldots ,n\right)$$
**Step** **3.** Determine the weighted decision matrix $R={\left[r_{ij}\right]}_{m\times n}$ by Equation (9).
  (9) $$r_{ij}=w_{j}d_{ij}=\sqrt{1-{\left(1-\mu_{ij}^{2}\right)}^{w_{j}},\nu_{ij}^{2}},\;\left(j=1,2,\ldots ,n\right)$$
**Step** **4.** Calculate the score function $s\left(r_{ij}\right)$ for all (i=1,2,…,m) and (j=1,2,…,n).
  (10) $$s\left(p\right)=\mu^{2}-\nu^{2}$$
**Step** **5.** Determine the maximizing $s(P_{i})$ and minimizing $s(R_{i})$ index as follows:
  (11) $$s\left(P_{i}\right)=\frac{1}{\left|B\right|}\sum_{j\in B}s(r_{ij})$$
  (12) $$s\left(R_{i}\right)=\frac{1}{\left|NB\right|}\sum_{j\in NB}s(r_{ij})$$
  where *B* is the set of benefit criteria and NB is the set of all non-benefit criteria, for all (i=1,2,…,m).
**Step** **6.** Calculate the relative weight of each alternative $Q_{i}\left(i=1,2,\ldots ,m\right)$.
  (13) $$Q_{i}=s\left(P_{i}\right)+\frac{\sum_{i=1}^{n}e^{s(R_{i})}}{s\left(P_{i}\right)\sum_{i=1}^{n}\frac{1}{e^{s(R_{i})}}}$$
**Step** **7.** Determine the priority order $P_{r_{i}}\left(i=1,2,\ldots ,m\right)$.
  (14) $$P_{r_{i}}=\frac{Q_{i}}{maxQ_{i}}\times 100$$
**Step** **8.** Rank the alternatives $A_{i}>A_{k}$ if $P_{r_{i}}\geq P_{r_{k}}$ for all (i,k=1,2,…,m).

## 6. Comparison with Proposed Entropies

When introducing newly developed methods, it is essential to conduct a comprehensive benchmarking analysis with a set of carefully selected reference methods. For this purpose, it is necessary to select an appropriate number of well-established benchmark methods that have been used for many years in global research. In this case, the role of reference methods is fulfilled by other typologies of entropy such as intuitionistic fuzzy entropies and Pythagorean fuzzy entropies proposed and adopted in other research [3,49]. The analysis performed serves to validate the results and reliability of new methods. It is critical to assess whether the results given by the newly proposed method are comparable to those provided by the reference methods and whether there are no significant outliers [15]. An objective measure of the consistency of the results provided by the compared methods is to measure the correlation between them. In this case, the Pearson correlation coefficient was used for this purpose. Due to mentioned facts, we first review various proposed entropies and draw a comparison example.

1. The IF entropy proposed by Zhang and Jiang [58].
  (15) $$\begin{array}{c} E_{1}=\frac{-1}{n}\sum_{i=1}^{n}\left[\frac{\mu_{A}\left(x_{i}\right)+1-\vartheta_{A}\left(x_{i}\right)}{2}log\left(\frac{\mu_{A}\left(x_{i}\right)+1-\vartheta_{A}\left(x_{i}\right)}{2}\right)\right.\\ \left.+\frac{\vartheta_{A}\left(x_{i}\right)+1-\mu_{A}\left(x_{i}\right)}{2}log\left(\frac{\vartheta_{A}\left(x_{i}\right)+1-\mu_{A}\left(x_{i}\right)}{2}\right)\right]. \end{array}$$
2. The Pythagorean fuzzy entropy introduced by Xue, Xu, Zhang, and Tian in 2017 [10].
  (16) $$E_{2}=\frac{1}{n}\sum_{i=1}^{n}\left[1-\left(\mu_{A\;}^{2}\left(x_{i}\right)+\vartheta_{A}^{2}\left(x_{i}\right)\right)\;\left|\mu_{A}^{2}{\left(x_{i}\right)-\vartheta }_{A}^{2}\left(x_{i}\right)\right|\right].$$

This entropy is used for deciding the investment in railway projects. This entropy is based on the linear programming technique for multi-dimensional analysis for preference (linmap) method.

3. Ye put forward two intuitionistic fuzzy entropy, which was further simplified by Wei et al. [59].
  The entropy proposed by Wei et al. [59] is:
  (17) $$E_{3}=\;\frac{1}{n}\sum_{i=1}^{n}\left[\left(\sqrt{2}cos\frac{\mu_{A}\left(x_{i}\right)-\vartheta_{A}\left(x_{i}\right)}{4}\pi -1\right)\times \frac{1}{\sqrt{2}-1}\right].$$
4. The following entropy was put forward by Manseng Liu and Haiping Ren [60]:
  (18) $$E_{4}=\frac{1}{n}\sum_{i=1}^{n}cot\left(\frac{\pi }{4}+\frac{\left|\;\mu_{A}\left(x_{i}\right)-\vartheta_{A}\left(x_{i}\right)\right|\times \left(1-\pi_{A}\left(x_{i}\right)\right)}{4}\pi \right).$$
5. The following entropy was developed by Wang and Wang [57]:
  (19) $$E_{5}=\frac{1}{n}\sum_{i=1}^{n}cot\left(\frac{\pi }{4}+\frac{|\mu_{A}\left(x_{i}\right)-\vartheta_{A}\left(x_{i}\right)|}{4(1+\pi_{A}\left(x_{i}\right))}\pi \right).$$

In order to compare entropies between each other, we decided to use Pearson’s correlation coefficient. The Pearson correlation coefficient compares two data sets using covariance and standard deviation [61]. Its value ranges from −1 to 1. The smaller the Pearson correlation coefficient value, the less correlation between the data, while the more significant the value, the greater the correlation. Formula (20) can represent it.

(20) $$r\left(x,y\right)=\frac{\sum_{i=1}^{n}\left(x_{i}-\bar{x}\right)\left(y_{i}-\bar{y}\right)}{\sqrt{\sum_{i=1}^{n}{\left(x_{i}-\bar{x}\right)}^{2}}\sqrt{\sum_{i=1}^{n}{\left(y_{i}-\bar{y}\right)}^{2}}}$$

## 7. Practical Case Studies

This section provides and discusses two illustrative examples of using Pythagorean fuzzy entropy. The first example deals with determining entropy values for Pythagorean fuzzy sets according to 6 methods. The second example shows the practical application of the proposed entropy in obtaining weights in a multi-criteria problem.

**Example** **1.** *Firstly, we will draw a table in which we will compare various entropies proposed by other researchers. Further, we will propose an example of the complex proportional assessment (COPRAS) method used for problems of multi-criteria decision making. This method is based on the new entropy proposed (new fuzzy entropy on Pythagorean fuzzy sets). This study used the uncertain decisions library available at https://gitlab.com/kiziub/uncertain-decisions (accessed on 10 November 2021 ).*

*Suppose $M=(x_{i},\mu_{M}\left(x_{i}\right),\vartheta_{M}\left(x_{i}\right)|\;x_{i}\epsilon X$, where X is the universal set be a Pythagorean fuzzy set for every real number [62].*

*Let $M_{1}=<0.5,0.5>,M_{2}=<0.1,0.9>$ and $M_{3}=<0.7,0.3>$.*

*From Table 1, we conclude that whenever μ=ϑ, the entropy reaches a maximum. The entropy attains a maximum value at $M_{1}$.*

**Example** **2.** *Assume that we have to choose one express company out of four express companies $\left\{x_{1},x_{2},x_{3},x_{4}\right\}$. After successful evaluation, the four alternatives with reference to four criteria are being decided. The decided criteria are $y_{1}$ for convince, $y_{2}$ for safety, $y_{3}$ for reliability, $y_{4}$ for tangibility where $\left\{y_{1},y_{2},y_{3}\right\}$ are the benefit criteria and $\left\{y_{4}\right\}$ is non-benefit criteria [15].*

*We consider this problem with the proposed COPRAS method. The various steps are as follows:*

***Step*** ***1.*** *Consider the criteria $Y_{j}\left(j=1,2,3,4\right)$ is the Pythagorean fuzzy set on the alternatives of set $X=\{x_{1},x_{2},x_{3},x_{4}\}$ presented by Table 2. With the help of Equation (7), find entropy $e_{1}=0.4846,e_{2}=0.2226,e_{3}=0.3623,e_{4}=0.6815$.*
***Step*** ***2.*** *With the help of Equation (8), find the weights of all criteria $\omega_{1}=0.2292,\omega_{2}=0.3457,\omega_{3}=0.2835,\omega_{4}=0.1416$, where $e_{j}$ is calculated using the selected Pythagorean fuzzy entropy.*
***Step*** ***3.*** *Compute the decision matrix $Z={\left[z_{i\;j}\right]}_{m\times n}$ where $z_{i\;j}=\left(\mu_{ij},\vartheta_{ij}\right)$ which is represented by Table 3.*
***Step*** ***4.*** *With the help of Equation (2), find the score function $s(a_{ij})\;\forall \;i,j=1,2,3,4$. The function values obtained are shown in Table 4.*
***Step*** ***5.*** *Find $S(P_{i})$ and $S(R_{i})\forall \;i=1,2,3,4$ with help of Equations (11) and (12).*
***Step*** ***6.*** *Determine the relative weight for every alternative $Q_{i}$ where i=1,2,3,4.*
***Step*** ***7.*** *Calculate the priority order ${Pr}_{i}$ where i=1,2,3,4, using Equation (14).*
***Step*** ***8.*** *Provide the ranking of alternatives with reference to the priority order.*

*The rankings of the alternatives obtained by the COPRAS method using each entropy method are displayed in Table 5. The obtained weights with the proposed entropy method gave the same ranking of the alternatives in the COPRAS method as the entropy method of Xue, Wang, Liu, and Zhang. However, the obtained weights for Wei’s entropy method resulted in a different ranking than the proposed method.*

**Figure 2:**
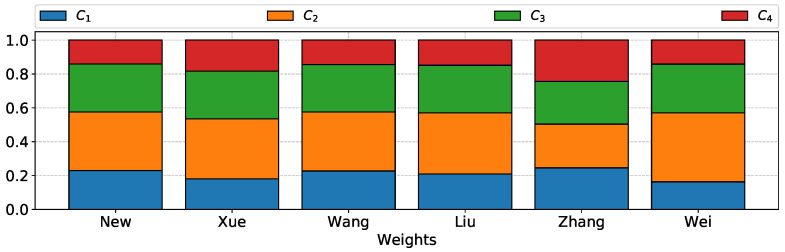
Weights determined by entropy methods for each criterion.

*The weights of the entropy methods for each criterion are visualized in Figure 2. By most of the methods, the least weight was assigned to criterion four. However, the entropy method proposed by Xue assigned the least weight to criterion one. Criterion two turned out to be the most significant criterion for all methods of selecting weights using entropy. The least varied weight values were obtained by Zhang’s entropy-based weight selection method.*

**Figure 3:**
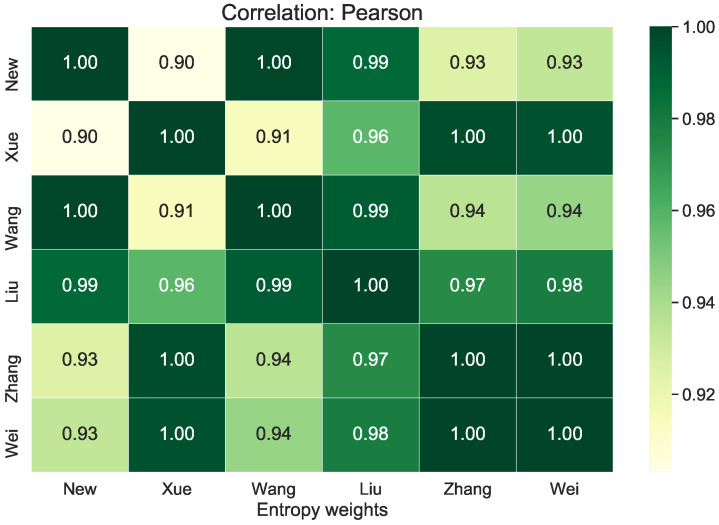
Pearson correlation heat maps for different entropy weights methods.

*Figure 3 displays the for each entropy method used to select the weights. All the considered methods have a high correlation with each other. However, the proposed method has the highest correlation with the Wang (1.00) and Liu (0.99) methods. On the other hand, it has the lowest correlation with the Xue method (0.90). Xue’s method has the highest correlation with Zhang’s method and Wei’s method, where the Pearson correlation coefficient value for this comparison was 1.00. One of the lower Pearson correlation values was achieved for Wang’s method and Xue’s method and was 0.91.*

## 8. Conclusions

This paper introduces the concept of the fuzzy entropy of Pythagorean fuzzy sets, which extends the concept of the fuzzy entropy of the intuitionistic fuzzy sets. As indicated in the article, this extension is necessary. It exploited the concept of probability for determining the fuzzy entropy of Pythagorean fuzzy sets. We gave some numerical illustrations to analyze our proposed entropy measure compared to some current entropies of Pythagorean fuzzy sets. The numerical illustrations show that the proposed entropy measures seem to be more reliable for exhibiting the degree of fuzziness of a PFS. We also proposed a COPRAS multi-criteria decision-making method with weights calculated based on the proposed new entropy measure. The illustrated numerical example demonstrates that the calculated results according to the proposed new method are similar to the calculation results according to some other existing methods.

The innovative integrated approach proposed by the authors, combining Pythagorean fuzzy entropy for Pythagorean fuzzy sets with the COPRAS method for multi-criteria evaluation of a problem with uncertain data, enables one to deal with the limitations that occur when using methods that are subjective or that do not take into account uncertainties in the data, which could make the final results inaccurate. The most significant positive outcomes of the approach presented in this article are:

- Adopting a fuzzy number environment that reflects well the uncertainty in the problems being solved;
- Resistance to uncertainties and inaccuracies appearing in the evaluated problem;
- High accuracy of delivered results confirmed by outcomes provided by reference methods.

However, further research with other complex data sets and problems is required to identify potential disadvantages and shortcomings of the proposed method and to identify possible needed further directions to improve or extend this approach. The proposed approach could be applied to problems associated with determining the risk of systems prior to implementation, such as the one presented in [49].

## Figures and Tables

**Table 1:** Comparison of the degree of fuzziness with different entropy measures.

Entropy	$E_{1}$	$E_{2}$	$E_{3}$	$E_{4}$	$E_{5}$	E
$M_{1}$	0.3010	1	1	1	1	1
$M_{2}$	0.1411	0.344	0.3479	0.1583	0.1583	0.0223
$M_{3}$	0.2652	0.768	0.8328	0.5095	0.5095	0.2360

**Table 2:** The Pythagorean fuzzy matrix given by contractor.

Z	$Y_{1}$	$Y_{2}$	$Y_{3}$	$Y_{4}$
$x_{1}$	0.2, 0.4	0.7, 0.2	0.8, 0.1	0.5, 0.5
$x_{2}$	0.8, 0.5	0.6, 0.3	0.1, 0.5	0.8, 0.2
$x_{3}$	0.3, 0.1	0.8, 0.05	0.7, 0.6	0.7, 0.6
$x_{4}$	0.1, 0.6	0.3, 0.7	0.4, 0.7	0.4, 0.4

**Table 3:** The weighted Pythagorean fuzzy matrix given by contractor.

Z	$Y_{1}$	$Y_{2}$	$Y_{3}$	$Y_{4}$
$x_{1}$	0.0965, 0.8105	0.4557, 0.5732	0.5014, 0.5206	0.1998, 0.9065
$x_{2}$	0.4569, 0.8531	0.3781, 0.6595	0.0533, 0.8216	0.3670, 0.7962
$x_{3}$	0.1462, 0.5899	0.5455, 0.3550	0.4169, 0.8651	0.3016, 0.9302
$x_{4}$	0.0480, 0.8895	0.1791, 0.8840	0.2196, 0.9038	0.1562, 0.8783

**Table 4:** The score functions and ranking of alternatives.

S	$Y_{1}$	$Y_{2}$	$Y_{3}$	$Y_{4}$	$P_{I}$	$R_{i}$	$Q_{i}$	$\frac{100Q_{I}}{\max Q_{i}}$	Rank
$x_{1}$	−0.6477	−0.1210	−0.0196	−0.7818	−0.2628	−0.7818	0.2758	94.70	2
$x_{2}$	−0.5190	−0.2920	−0.6722	−0.4992	−0.4944	−0.4992	−0.0884	−30.35	3
$x_{3}$	−0.3266	0.1715	−0.5748	−0.7743	−0.2433	−0.7743	0.2913	100	1
$x_{4}$	−0.7889	−0.7494	−0.7687	−0.7470	−0.7690	−0.7470	−0.2488	−85.42	4

**Table 5:** The comparison with some other methods.

$x_{i}$	Proposed	Xue, Xu,	Wang and	Liu and	Zhang and	Wei
	Entropy	and Zhang [10]	Wang [57]	Ren [60]	Jiang [58]	[59]
$x_{1}$	2	2	2	2	2	1
$x_{2}$	3	3	3	3	3	3
$x_{3}$	1	1	1	1	1	2
$x_{4}$	4	4	4	4	4	4

## Abbreviations

The following abbreviations are used in this manuscript:

AHP	Analytic hierarchy process
COMET	Characteristic objects method
COPRAS	Complex proportional assessment
CRITIC	Criteria importance through inter-criteria correlation
ELECTRE	Elimination and choice expressing the reality
FS	Fuzzy set
IFS	Intuitionistic fuzzy sets
MACBETH	Measuring attractiveness by a categorical based evaluation technique
MAUT	Multi-attribute utility theory
MCDA	Multi-criteria decision analysis
MCDM	Multi-criteria decision making
NAIADE	Novel approach to imprecise assessment and decision environments
PAPRIKA	Potentially all pairwise rankings of all possible alternatives
PFE	Pythagorean fuzzy entropy
PFS	Pythagorean fuzzy set
PROMETHEE	Preference ranking organization method for enrichment evaluations
SMART	Simple multi-attribute rating technique
TACTIC	Treatment of the alternatives according to the importance of criteria
TOPSIS	Technique for order of preference by similarity to ideal solution
VIKOR	Vise kriterijumska optimizacija i kompromisno resenje
